# Sternocleidomastoid muscle morphology is independently associated with neck strength and endurance in athletes and para-athletes

**DOI:** 10.3389/fphys.2026.1852707

**Published:** 2026-08-13

**Authors:** Sandra Rozenstoka, Elena Comadran de Barnola, Santa Kauzena, Gundega Akuratere, Zinta Galeja, Daira Deicmane

**Affiliations:** 1 Institute of Public Health, Riga Stradins University (RSU), Riga, Latvia; 2 Sports Laboratory Clinic – FIMS Collaborating Centre of Sports Medicine, Riga, Latvia; 3 Latvian Sports Medicine Association (LSMA), Riga, Latvia; 4 European Federation of Sports Medicine Associations (EFSMA), Lausanne, Switzerland; 5 International Federation of Sports Medicine (FIMS), Lausanne, Switzerland; 6 EXER-GENUD (Growth, Exercise, NUtrition and Development) Research Group (S72_23R), FIMS Collaborating Centre of Sports Medicine, University of Zaragoza, Zaragoza, Spain

**Keywords:** anthropometry, injury prevention, neck endurance, neck strength, sports medicine, sternocleidomastoid, ultrasound

## Abstract

**Background:**

Neck functional capacity is clinically relevant in sports medicine for performance support, screening, and injury prevention in sports characterized by acceleration, contact, and sustained precision demands. Ultrasound can quantify local muscle morphology and may improve interpretation of neck performance beyond crude anthropometry (e.g., neck girth) and global strength indicators.

**Objective:**

To determine whether sternocleidomastoid (SCM) cross-sectional area (CSA) and neck anthropometry are associated with neck function (strength, endurance, range of motion), and whether SCM morphology explains function beyond age, BMI, general strength (handgrip), and sport-domain context in a male cohort of athletes, para-athletes, and physically active controls.

**Methods:**

Cross-sectional comparative study (n=195 men) including athletes across sport domains, para-athletes, and controls. Outcomes were isometric neck strength (flexor and extensor), neck flexor endurance, and active neck ROM. SCM CSA was assessed by standardized musculoskeletal ultrasound; neck girth served as an anthropometric comparator. Analyses included Pearson correlations and adjusted linear regression models controlling for age and BMI. Explanatory value beyond general strength was evaluated by adding handgrip strength, and independence from sport-domain context by adding sport domain as a covariate.

**Results:**

SCM CSA correlated positively with flexor strength (r=0.34, p<0.001), extensor strength (r=0.25, p<0.001), and flexor endurance (r=0.28, p<0.001), and showed a weak inverse association with extension ROM (r=−0.17, p=0.017). In adjusted models (age, BMI), SCM CSA was independently associated with flexor strength (β=1.32 kg per cm²; p<0.001) and flexor endurance (β=0.53 min per cm²; p<0.001), but not extensor strength. SCM CSA remained associated with flexor strength and endurance after adjustment for handgrip strength and sport domain. Additional within- versus between-domain analysis supported a within-domain SCM association for flexor strength and endurance rather than a purely between-domain aggregation effect. Neck girth did not provide comparable independent explanatory value for strength or endurance outcomes.

**Conclusions:**

SCM morphology is independently associated with neck performance, particularly flexor strength and endurance. Ultrasound-based SCM assessment provides measurable but limited additional information beyond neck girth, handgrip strength, and sport-domain context. These findings support its cautious use as an adjunct measure alongside objective cervical functional testing, rather than as a stand-alone screening tool.

## 1 Introduction

Neck function is relevant to athletic performance and clinical management because neck musculature contributes to head–neck stabilization, gaze control, and tolerance to external loading during sport (Eckner et al., 2014; Patricios et al., 2023). This is particularly important in sports characterized by repeated contact (e.g., rugby, ice hockey, martial arts), high accelerative loads (e.g., luge, bobsleigh, motorsport), or prolonged precision postures (e.g., shooting, archery), where neck capacity may influence both task execution and the athlete’s response to mechanical stress (Eckner et al., 2014; Patricios et al., 2023). Contemporary sports medicine frameworks and sport-specific evidence therefore support objective assessment of neck function as part of athlete profiling, targeted conditioning, and head–neck risk management (Peek et al., 2020; Leung et al., 2022; Patricios et al., 2023; Alvacite Cardenas and Preston, 2025).

In practice, however, neck assessment often relies on crude anthropometric indicators such as neck circumference or visual appraisal of neck size (Esopenko et al., 2020). Although attractive because they are quick and inexpensive, these measures are biologically non-specific: neck girth reflects not only muscle mass, but also adipose and other non-contractile tissues. Recent work has questioned whether a larger neck can be assumed to represent greater functional neck capacity, suggesting that simple circumference-based measures may be an insufficient proxy for clinically meaningful neck performance (Esopenko et al., 2020; Garrett et al., 2023).

Ultrasound offers a feasible and non-invasive method to quantify local muscle morphology *in vivo* (Nagai et al., 2020; Sidiropoulos et al., 2024). For the cervical region, this is particularly appealing because it allows clinicians and researchers to move beyond gross anthropometry toward muscle-specific structural assessment (Nagai et al., 2020; Sidiropoulos et al., 2024). The sternocleidomastoid (SCM) is especially relevant in this context. It is superficial, anatomically accessible to ultrasound, and functionally involved in neck flexion and anterior head–neck stabilization (Eckner et al., 2014; Sidiropoulos et al., 2024). In addition, recent studies support the reliability of ultrasound-based assessment of neck muscle morphology under standardized protocols, strengthening its potential utility in sports medicine and performance settings (Nagai et al., 2020; Sidiropoulos et al., 2024).

Despite this rationale, an important gap remains. Most sports medicine discussions around the neck have focused on global strength, concussion-related risk, head-acceleration biomechanics, or broad conditioning strategies (Peek et al., 2020; Garrett et al., 2023; Patricios et al., 2023; Alvacite Cardenas and Preston, 2025), whereas less is known about the association between local neck muscle morphology and neck function across heterogeneous sport contexts. In particular, para-athletes are frequently excluded, pooled, or underpowered for separate analysis, despite potentially distinct cervical loading environments and impairment-related constraints. It is also unclear whether SCM CSA provides functional information beyond routine descriptors such as age, body size, or general strength. We included handgrip strength as a pragmatic proxy of global upper-limb strength to test whether SCM morphology provides neck-specific explanatory value beyond general strength capacity.

Therefore, the aims of this study were: (1) to quantify the associations between SCM CSA and neck function, including flexor and extensor strength, flexor endurance, and range of motion; (2) to compare SCM CSA with neck girth as predictors of neck function after adjustment for age and BMI; (3) to test whether SCM CSA remains associated with neck function after accounting for general strength using handgrip; and (4) to examine whether these associations persist after accounting for sport-domain context.

We hypothesized that SCM CSA would be more strongly associated with neck flexor strength and endurance than with other functional outcomes, would outperform neck girth as a structural proxy of neck function, and would remain independently associated with neck performance after adjustment for age, BMI, and handgrip strength.

## 2 Materials and methods

### 2.1 Study design

This cross-sectional study is embedded within a broader comparative program “The Impact of Sports and Para Sports on Neck Functional Capacity, Stereotype of Movements and Health Risk”.

Reporting follows the STROBE guideline for observational studies.

The study was conducted in accordance with the Declaration of Helsinki. Ethical approval for this study was obtained from the Ethics Committee of the Latvian Sports Medicine Association, Latvia (Ref: E – 3, 22.04.2024). All participants were adults and provided written informed consent before enrolment.

### 2.2 Participants

Participants were assessed at the Sports Laboratory Clinic – FIMS Collaborating Centre of Sports Medicine (Riga, Latvia) and affiliated RSU facilities. Athletes and para-athletes were recruited through collaborating sports federations, clubs, and national-team medical networks; controls were physically active men recruited from the community and university networks. Eligibility criteria included: male sex; age ≥18 years; regular sport participation (athletes/para-athletes) or regular physical activity (controls); and ability to complete the neck testing protocol. Exclusion criteria included acute neck injury at the time of testing, recent cervical surgery, or any medical condition precluding maximal isometric effort. The full cohort comprised 195 participants across five sport domains (Acceleration n = 34, Contact n = 63, Targeting n = 16, Para-athletes n = 20, Control n = 62), which comprised a total of 12 sports (**Table 1**). Three sports with fewer than five participants each (carsport, rugby, and target-curling) were excluded from all analyses prior to statistical modelling. Para-athletes included wheelchair basketball players, wheelchair fencing, wheelchair curling, para-archery, para-shooting and para-bobsleigh, classified as an independent domain irrespective of their sport code. All primary morphology–function analyses were conducted on the full sample of 195 participants. Of note, one para-athlete had an upper-limb amputation and could not perform the handgrip test, which reduced the sample size for this specific test to 194 participants. Detailed demographic and sport-background characteristics are presented in **Table 2**.

**Table 1:** Sport domains and subgroups (n = 195).

Sport domain/subgroup	n	Disciplines included
**Acceleration**	**34**	
Sliding	17	Bobsleigh (n = 7), luge (n = 7), skeleton (n = 3)
Motorsports	17	Motorsports
**Contact**	**63**	
Basketball	14	Basketball
Boxing	11	Boxing
Martial Arts	15	Martial arts (jiu jitsu, wrestling)
Soccer	10	Football/soccer
Ice Hockey	13	Ice hockey
**Targeting**	**16**	
Shooting	16	Shooting (n = 11), archery (n = 5)
**Para-athletes**	**20**	
Wheelchair basketball	9	IWF sports classes 2–3.5
Para archery	5	Classified/eligible
Shooting para sports	2	Classified/eligible
Wheelchair curling	2	Classified/eligible
Wheelchair fencing	1	Class B
Para bobsleigh	1	Classified/eligible
**Control**	**62**	
Cycling	20	Recreational/fitness cycling
Fitness	14	Gym-based fitness training
Running/Motorsports	28	Running (n = 13), recreational motorsports (n = 15)
**Total**	**195**	*5 domains · 12 subgroups*

† Three athlete subgroups with n < 5 were excluded from all statistical analyses (Carsport n = 3, Rugby n = 4, Target-curling n = 4). All para-athletes retained.

Bold values indicate sport-domain totals/headings. Non-bold rows indicate the corresponding subgroups and disciplines within each domain.

**Table 2:** Demographic and sport-background characteristics by sport domain (mean ± SD for continuous variables; n (%) for categorical).

Variable	Total (n = 195)	Acceleration (n=34)	Contact (n=63)	Targeting (n=16)	Para-athlete (n=20)	Control (n=62)
Anthropometric and physiological characteristics						
Age (yr)	33.5 ± 9.4	29.6 ± 10.4	27.7 ± 8.1	39.1 ± 7.1	38.5 ± 7.7	38.3 ± 6.5
Weight (kg)	83.7 ± 10.5	84.1 ± 10.9	84.6 ± 10.8	85.9 ± 9.5	76.2 ± 11.4	84.6 ± 9.0
Height (cm)	182.3 ± 6.9	182.4 ± 7.1	183.8 ± 7.3	182.1 ± 5.2	177.2 ± 7.3	182.2 ± 6.0
BMI (kg/m²)	25.2 ± 2.6	25.2 ± 2.0	25.0 ± 2.7	25.9 ± 2.5	24.3 ± 3.7	25.5 ± 2.3
Skeletal muscle mass (kg) †	39.2 ± 4.2‡	39.3 ± 4.7	39.9 ± 4.5	38.1 ± 3.2	n/a†	38.8 ± 3.8
Body fat (%) †	17.8 ± 5.9‡	16.9 ± 5.2	16.3 ± 5.8	20.9 ± 7.4	n/a†	19.0 ± 5.5
Years in specific sport	11.6 ± 6.6	10.3 ± 6.8	15.4 ± 6.6	13.9 ± 4.2	11.4 ± 5.1	8.0 ± 5.2
Sport sessions per week	3.4 ± 1.6	3.2 ± 1.6	4.6 ± 1.5	2.9 ± 1.4	2.8 ± 1.1	2.6 ± 1.2
Sport training (min/week)	392 ± 230	422 ± 198	451 ± 231	442 ± 221	392 ± 243	304 ± 225
Sex						
Sex, male	195 (100%)	34 (100%)	63 (100%)	16 (100%)	20 (100%)	62 (100%)
Competition level (last 3 years), n (%)						
International/World/European/Olympic	106 (54%)	27 (79%)	35 (56%)	10 (62%)	19 (95%)	15 (24%)
National championship	74 (38%)	7 (21%)	28 (44%)	6 (38%)	1 (5%)	32 (52%)
No competition	15 (8%)	0	0	0	0	15 (24%)
Training period, n (%)						
Pre-season	110 (56%)	18 (53%)	31 (49%)	10 (62%)	13 (65%)	38 (61%)
In-season	85 (44%)	16 (47%)	32 (51%)	6 (38%)	7 (35%)	24 (39%)
Training characteristics						
Neck-specific exercises included, n (%)	55 (28%)	11 (32%)	18 (29%)	8 (50%)	2 (10%)	16 (26%)

BIA, bioelectrical impedance analysis.

†Skeletal muscle mass and body fat assessed by BIA. BIA was not feasible for most para-athletes using wheelchairs; para-athlete values are excluded (n/a). ‡ Athletes only (n = 175).

### 2.3 Sport-domain classification

Participants were classified into sport domains based on the predominant biomechanical and loading characteristics of their sport (**Table 1**): Acceleration (sustained G-forces and vibration; Sliding, Motor subgroups), Contact (repeated impact and collision loads; Basketball, Boxing, Martial Arts, Soccer, Ice Hockey), Targeting (sustained precision neck posture; Shooting), Para-athletes (diverse impairment profiles; upper-limb ergometry standard), and Control (physically active non-collision individuals; Cycling, Fitness, Running/Motorsports).

### 2.4 Anthropometry and neck functional capacity assessments

All functional assessments were conducted in a standardized sequence by the same trained examiner. The following outcomes were used in the present paper:

Anthropometry assessments: Body mass index (BMI) was derived from height and weight measured by stadiometer and calibrated scales; body composition (skeletal muscle mass, body fat percentage) was assessed by bioelectrical impedance analysis (BIA).

Isometric neck strength: maximum isometric neck flexor strength (kg) was measured in the supine lying position using a portable digital hand-held fixed dynamometer (EasyForce, Meloq AB, Sweden), and is a primary outcome. Extensor strength (kg) was measured in the prone position using the same protocol, and is a secondary outcome. The lying position was selected to isolate cervical musculature and minimize compensatory trunk contribution. Maximum values are reported. Published cervical dynamometry studies support the reliability of hand-held or stabilized hand-held dynamometry protocols for isometric neck strength assessment (Versteegh et al., 2015; Vannebo et al., 2018).

Neck flexor endurance (primary outcome): The timed supine head-lift test was employed, for which participants had to maintain a chin-tucked cervical flexion position against gravity for the maximum possible duration. The test has been used in neck endurance profiling and was applied consistently using the same standardized protocol across all participants; reliability and normative data for clinical deep neck flexor endurance testing have been reported previously (Olson et al., 2006; Domenech et al., 2011).

Active ROM (secondary outcome): Active neck ROM was measured using a digital goniometer (EasyAngle, Meloq AB, Sweden) in a standardized seated position. Six directions were assessed: flexion, extension, bilateral lateral flexion, and bilateral rotation.

Handgrip strength (specificity control): Handgrip strength (right hand) measured using a calibrated hand dynamometer (standard clinical protocol) and recorded in kilograms. This test was included as a proxy for general upper-limb strength in structural specificity models.

### 2.5 Neck morphological assessments

SCM morphology (primary exposure): SCM CSA was assessed by musculoskeletal ultrasonography using a standardized supine protocol (knees bent, arms crossed on the chest, head and neck neutral, with the forehead and chin horizontal and centered). B-mode imaging was performed bilaterally in the axial/short-axis (transverse) plane using a high-frequency linear transducer (Clarius Scanner L15 HD3, Clarius Mobile Health, Canada; 15 MHz). Images were obtained at the level of the common carotid artery immediately proximal to the carotid bifurcation, or as close as possible to this anatomical level according to the individual acoustic window.

For total SCM CSA measurement, the full visible SCM cross-sectional boundary was manually traced along the inner echogenic fascial boundary, and the enclosed area (cm²) was used for analysis. When the SCM exceeded the ultrasound field of view, two adjacent transverse images were obtained by sliding the transducer laterally across the muscle. Anatomical landmarks were used to maintain spatial orientation and avoid overlap or omission between the adjacent image fields, and the partial CSA measurements were combined to obtain the total SCM CSA. At the level of the distal SCM division, the clavicular and sternal heads were measured separately using the same planimetric tracing approach.

**Figure 1:**
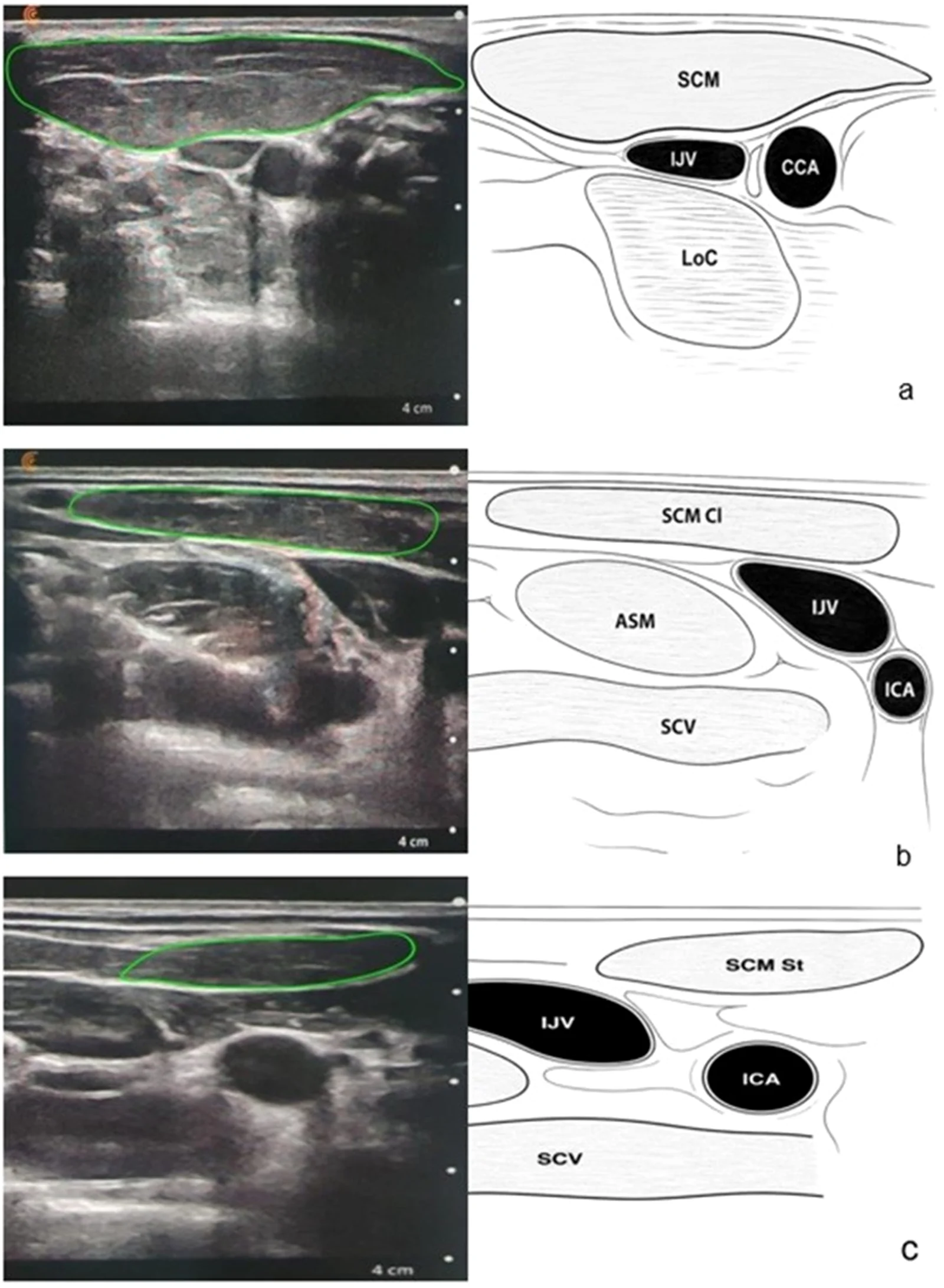
Representative axial/short-axis B-mode ultrasound images and corresponding schematic anatomical drawings of sternocleidomastoid (SCM) muscle assessment. **(A)** shows the SCM at the level of the common carotid artery (CCA), immediately proximal to the carotid bifurcation, with manual tracing of the full visible cross-sectional boundary for CSA measurement. Panel A represents a case in which the full SCM cross-section was visualized within a single ultrasound field of view; for wider muscles, two adjacent transverse images were acquired as described in the Methods. **(B, C)** illustrate separate assessment of the clavicular head (SCM Cl) and sternal head (SCM St), respectively, at the level of the distal SCM division. Green contours indicate the manually traced muscle boundary used for planimetric measurement. The schematic drawings are provided to clarify anatomical orientation and adjacent structures. SCM, sternocleidomastoid muscle; SCM Cl, clavicular head of the SCM; SCM St, sternal head of the SCM; IJV, internal jugular vein; CCA, common carotid artery; ICA, internal carotid artery; LoC, longus colli muscle; ASM, anterior scalene muscle; SCV, subclavian vein. Ultrasound system: Clarius Scanner L15 HD3 (Clarius Mobile Health, Canada).

The following anatomical measurements were recorded for each side: longitudinal width (mm), transverse height (mm), circumference (mm), and CSA (cm²) (**Figure 1**). Right total SCM CSA (cm²) was used as the primary morphological predictor to maximize measurement completeness and avoid collinearity with bilateral averages; the bilateral average and head-specific CSA values are reported descriptively.

### 2.6 Neck anthropometry (comparator exposure)

Neck girth (circumference, cm) was measured at the sub-chin level as an anthropometric comparator. Neck girth reflects a composite of muscle, adipose, and connective tissue and is commonly used in clinical screening as a proxy for neck size.

### 2.7 Statistical analysis

**Figure 2:**
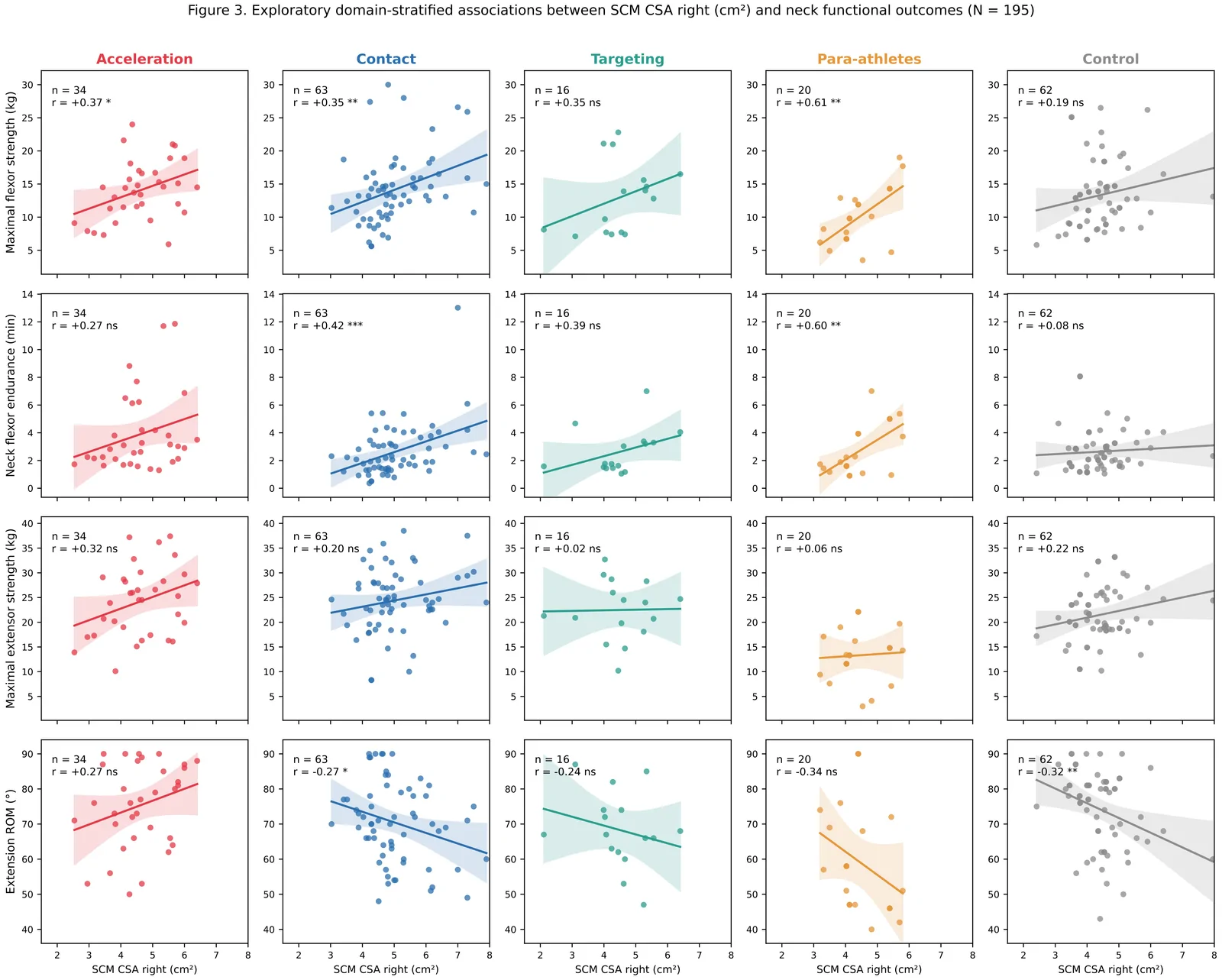
Exploratory domain-stratified associations between SCM CSA right (cm²) and neck functional outcomes (N = 195). Each panel shows the bivariate OLS regression line ±95% CI with Pearson r and n annotated. Rows (top to bottom): maximal flexor strength (kg), neck flexor endurance (min), maximal extensor strength (kg), and extension ROM (°). Columns: Acceleration, Contact, Targeting, Para-athletes, and Control. The figure is descriptive and is used to visualize within-domain patterns; formal inference is based on the pre-specified regression models and *post-hoc* decomposition analysis.

All primary analyses used Pearson correlations and ordinary least squares (OLS) regression, given that continuous outcome variables were normally distributed based on Shapiro–Wilk testing and inspection of Q–Q plots. Residual plots were also inspected to assess model assumptions. All primary morphology–function analyses used n = 195 participants; models including handgrip strength used n = 194 because one para-athlete was unable to perform the handgrip test. Group comparisons for domain-wise descriptive analyses used Kruskal–Wallis tests. A p-value < 0.05 was considered statistically significant throughout. For the domain-stratified bivariate plots (**Figure 2**), within-domain correlations are presented for descriptive visualization and should be interpreted as exploratory; formal inference is based on the pre-specified primary outcomes and multivariable models.

Step 1 — Bivariate correlations: Pearson correlation coefficients (r) were calculated between SCM CSA right and (i) maximal neck flexor strength, (ii) maximal extensor strength, (iii) neck flexor endurance, and (iv) neck extension ROM. Neck flexor strength and neck flexor endurance were predefined primary outcomes, based on the *a priori* mechanistic role of SCM in anterior stabilization and flexion. Correlations with extensor strength and extension ROM were treated as secondary/exploratory. The same analyses were performed for neck girth. Sample size (n) is reported for each correlation pair.
Step 2 — Adjusted regression models: OLS regression models were fitted sequentially to examine whether SCM CSA was associated with each cervical outcome. All models included age and BMI as core covariates because of their established associations with muscle morphology and functional capacity. Model 1a included SCM CSA, age, and BMI. Model 1b replaced SCM CSA with neck girth as an anthropometric comparator to evaluate whether ultrasound-based morphology provided explanatory value beyond clinically accessible circumference measures. Model 2 additionally included right-hand handgrip strength to test whether SCM CSA contributed to neck function beyond general upper-limb strength. Model 3 further included sport domain as a five-level categorical covariate (reference = Control) to account for sport-specific loading context. Because para-athletes formed an exclusive domain in the classification, a separate para-athlete status variable was not entered together with sport domain to avoid collinearity. Model R², adjusted R², sample size, regression coefficients, 95% confidence intervals, and p-values are reported where relevant.

A *post-hoc* exploratory within- versus between-domain decomposition was performed for the primary SCM models. SCM CSA was separated into a domain-mean component (between-domain SCM CSA) and an individual deviation from the domain mean (within-domain, group-mean-centered SCM CSA); both components were entered simultaneously with age and BMI. Sport domain was not entered as an additional categorical term in this decomposition because the between-domain component represented the domain-level SCM mean. In addition, *post-hoc* exploratory para-athlete versus non-para comparisons were performed for the four key cervical functional outcomes using Mann–Whitney U tests, with Holm adjustment across the four comparisons.

## 3 Results

### 3.1 Participant characteristics

A total of 195 athletes and para-athletes were included in all primary morphology–function analyses (Acceleration n = 34, Contact n = 63, Targeting n = 16, Para-athletes n = 20, Control n = 62); detailed demographic and sport-background characteristics are presented in **Table 2**. One para-athlete was unable to perform handgrip testing due to upper-limb amputation, reducing the specificity model sample to n = 194. Morphological and functional characteristics by domain are presented in **Table 3**.

**Table 3:** Morphological and functional characteristics by sport domain (mean ± SD).

Variable	Total (n = 195)	Acceleration(n=34)	Contact (n=63)	Targeting (n=16)	Para-athlete (n=20)	Control (n=62)	H	p
Morphological variables								
Neck girth (cm)	39.5 ± 2.8	38.6 ± 2.8	39.2 ± 3.0	40.2 ± 2.3	41.3 ± 3.1	39.4 ± 2.1	12.83	0.012
Neck width (cm)	12.1 ± 0.8	12.2 ± 0.7	12.1 ± 1.0	12.0 ± 0.8	12.5 ± 0.8	11.9 ± 0.7	9.28	0.054
Neck depth (cm)	12.0 ± 1.2	11.8 ± 1.0	11.8 ± 1.3	12.3 ± 1.2	13.4 ± 0.9	12.2 ± 1.1	27.58	<0.001
SCM CSA bilateral avg (cm²)	4.69 ± 1.03	4.83 ± 1.32	4.92 ± 0.94	4.60 ± 1.03	4.52 ± 0.84	4.47 ± 0.97	10.50	0.033
SCM CSA right (cm²)	4.67 ± 0.97	4.62 ± 0.96	5.05 ± 1.03	4.50 ± 1.00	4.42 ± 0.77	4.44 ± 0.86	12.00	0.018
SCM clavicular head avg (cm²)	1.81 ± 0.43	2.06 ± 0.31	1.90 ± 0.50	1.83 ± 0.53	1.73 ± 0.44	1.74 ± 0.38	9.10	0.058
SCM sternal head avg (cm²)	1.72 ± 0.53	1.59 ± 0.43	1.90 ± 0.52	1.72 ± 0.79	1.70 ± 0.52	1.60 ± 0.50	1.22	0.875
Functional outcomes								
Maximal flexor strength (kg)	13.4 ± 5.1	14.1 ± 4.4	14.2 ± 5.3	13.0 ± 5.4	10.0 ± 4.3	13.4 ± 5.1	12.60	0.013
Maximal extensor strength (kg)	22.2 ± 6.9	24.2 ± 7.0	24.5 ± 6.4	22.5 ± 6.0	13.3 ± 5.3	21.5 ± 5.4	39.38	<0.001
Neck flexor endurance (min)	2.86 ± 1.97	3.90 ± 2.77	2.64 ± 1.90	2.62 ± 1.61	2.67 ± 1.81	2.64 ± 1.44	8.36	0.079
Extension ROM (°)	71.1 ± 12.6	75.4 ± 12.0	70.4 ± 11.5	68.3 ± 10.8	59.4 ± 15.1	74.1 ± 11.1	21.67	<0.001

H = Kruskal–Wallis test statistic (df = 4). p = omnibus p-value across the five domains.

An exploratory *post-hoc* comparison between para-athletes and non-para participants was performed for the four key cervical functional outcomes. Compared with all other participants pooled, para-athletes had significantly lower maximal flexor strength (10.0 ± 4.3 vs 13.8 ± 5.1 kg; Holm-adjusted p = 0.003), markedly lower maximal extensor strength (13.3 ± 5.3 vs 23.2 ± 6.2 kg; Holm-adjusted p < 0.0001), and reduced extension ROM (59.4 ± 15.1° vs 72.5 ± 11.5°; Holm-adjusted p < 0.001). Neck flexor endurance did not differ between para-athletes and non-para participants (2.7 ± 1.8 vs 2.9 ± 2.0 min; Holm-adjusted p = 0.38).

### 3.2 Correlation analyses

**Figure 3:**
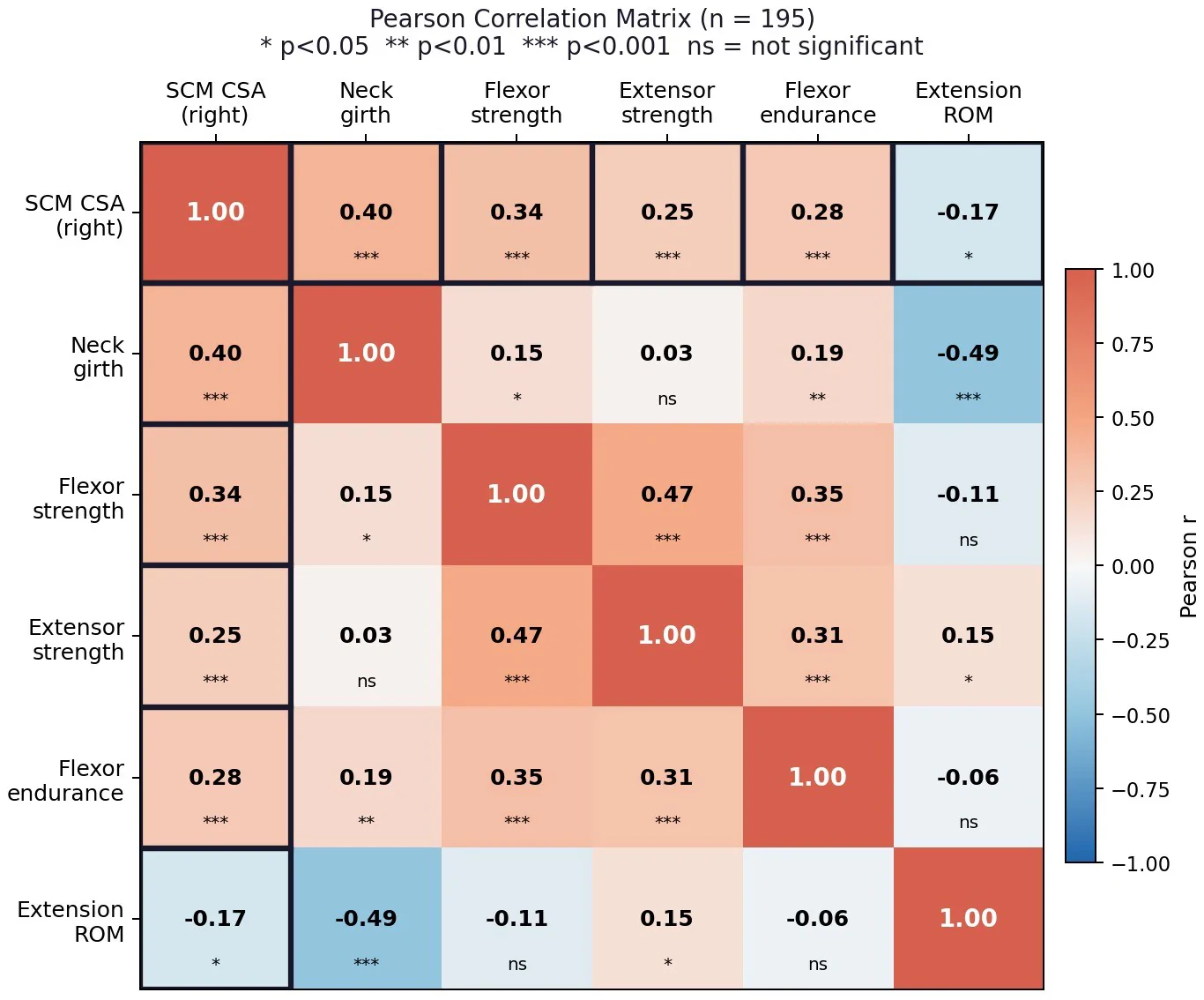
Pearson correlation matrix (n=195) for SCM CSA (right), neck girth, strength, endurance, and extension ROM. Cells show Pearson r with significance codes as indicated. The matrix is descriptive and complements the primary regression analyses.

SCM CSA right was significantly and positively correlated with maximal flexor strength and neck flexor endurance (primary outcomes), and also with maximal extensor strength (secondary outcome) (**Figure 3**). Correlations with maximal flexor strength and neck flexor endurance were considered primary; correlations with extensor strength and extension ROM were secondary/exploratory. The strongest association was with maximal flexor strength (r = 0.34, p < 0.001, n = 195), followed by neck flexor endurance (r = 0.28, p < 0.001) and maximal extensor strength (r = 0.25, p < 0.001). SCM CSA showed a weak inverse bivariate association with neck extension ROM (r = −0.17, p = 0.017), but this association was not significant after adjustment for sport domain in Model 3.

A Pearson correlation matrix summarizing SCM CSA (right), neck girth, and key functional outcomes is provided in **Figure 3** to complement the primary regression models.

### 3.3 Adjusted regression models

All models were adjusted for age and BMI as core covariates. Key model findings across specifications are summarized in **Table 4**, with regression coefficients, 95% confidence intervals, exact p-values, adjusted R², and semi-partial ΔR² reported for the primary predictor in each model. The results are described below by model family to distinguish SCM CSA effects from neck girth, general strength, and sport-domain context.

**Table 4:** Adjusted regression estimates for SCM CSA and neck girth associations with cervical functional outcomes across model specifications.

Outcome/Model	Term	β [95% CI]	p	Adj. R²	ΔR² (semi-partial)	Nested F test
Maximal flexor strength (kg)						
Model 1a: SCM CSA + age + BMI	SCM CSA	+1.316 [+0.559, +2.073]	<0.001	0.146	0.052	F=11.75, p<0.001 (n=195)
Model 1b: neck girth + age + BMI	Neck girth	+0.045 [−0.274, +0.363]	0.781	0.094	0.000	F=0.08, p=0.781 (n=195)
Model 2: SCM CSA + handgrip + age + BMI	SCM CSA	+1.245 [+0.499, +1.991]	0.001	0.167	0.047	F=10.84, p=0.001 (n=194)
Model 3: SCM CSA + sport domain + age + BMI	SCM CSA	+1.347 [+0.568, +2.126]	<0.001	0.156	0.051	F=11.64, p<0.001 (n=195)
Maximal extensor strength (kg)						
Model 1a: SCM CSA + age + BMI	SCM CSA	+0.808 [−0.190, +1.807]	0.112	0.173	0.011	F=2.55, p=0.112 (n=195)
Model 1b: neck girth + age + BMI	Neck girth	−0.315 [−0.723, +0.093]	0.129	0.172	0.010	F=2.33, p=0.129 (n=195)
Model 2: SCM CSA + handgrip + age + BMI	SCM CSA	+0.683 [−0.281, +1.648]	0.164	0.218	0.008	F=1.95, p=0.164 (n=194)
Model 3: SCM CSA + sport domain + age + BMI	SCM CSA	+0.690 [−0.268, +1.649]	0.157	0.289	0.007	F=2.02, p=0.157 (n=195)
Neck flexor endurance (min)						
Model 1a: SCM CSA + age + BMI	SCM CSA	+0.529 [+0.225, +0.832]	<0.001	0.073	0.056	F=11.80, p<0.001 (n=195)
Model 1b: neck girth + age + BMI	Neck girth	+0.094 [−0.033, +0.221]	0.146	0.027	0.011	F=2.13, p=0.146 (n=195)
Model 2: SCM CSA + handgrip + age + BMI	SCM CSA	+0.517 [+0.212, +0.822]	<0.001	0.072	0.054	F=11.20, p<0.001 (n=194)
Model 3: SCM CSA + sport domain + age + BMI	SCM CSA	+0.582 [+0.277, +0.887]	<0.001	0.127	0.064	F=14.17, p<0.001 (n=195)
Extension ROM (°)						
Model 1a: SCM CSA + age + BMI	SCM CSA	−2.009 [−3.849, −0.169]	0.032	0.166	0.020	F=4.64, p=0.032 (n=195)
Model 1b: neck girth + age + BMI	Neck girth	−2.094 [−2.793, −1.395]	<0.001	0.278	0.130	F=34.91, p<0.001 (n=195)
Model 2: SCM CSA + handgrip + age + BMI	SCM CSA	−2.177 [−3.989, −0.365]	0.019	0.198	0.023	F=5.61, p=0.019 (n=194)
Model 3: SCM CSA + sport domain + age + BMI	SCM CSA	−1.174 [−2.914, +0.567]	0.185	0.303	0.006	F=1.77, p=0.185 (n=195)

Values are unstandardized regression coefficients (β) with 95% confidence intervals for the primary structural predictor in each model. Model 1a included SCM CSA, age, and BMI; Model 1b included neck girth, age, and BMI; Model 2 included SCM CSA, handgrip strength, age, and BMI; and Model 3 included SCM CSA, sport domain, age, and BMI. Adjusted R² describes the explanatory value of the full model. ΔR² represents the semi-partial R² of the SCM CSA or neck girth term, indicating the unique variance explained by that predictor beyond the other covariates in the same model. The nested F-test provides the corresponding single-degree-of-freedom test for the added structural predictor.

#### 3.3.1 SCM CSA models (model 1a: SCM CSA + age + BMI)

Maximal flexor strength. Right SCM CSA was independently associated with maximal flexor strength (β = +1.316 kg per cm², 95% CI [+0.559, +2.073], p < 0.001; adjusted R² = 0.146). The semi-partial ΔR² for SCM CSA was 0.052, indicating that SCM CSA explained a small but measurable proportion of unique variance beyond age and BMI.

Maximal extensor strength. Right SCM CSA showed a positive bivariate association with maximal extensor strength, but the adjusted association was weaker and non-significant in Model 1a (β = +0.808 kg per cm², 95% CI [−0.190, +1.807], p = 0.112; adjusted R² = 0.173).

Neck flexor endurance. Right SCM CSA was independently associated with neck flexor endurance (β = +0.529 min per cm², 95% CI [+0.225, +0.832], p < 0.001; adjusted R² = 0.073; semi-partial ΔR² = 0.056).

Neck extension ROM. Right SCM CSA showed a weak inverse association with extension ROM in Model 1a (β = −2.009° per cm², 95% CI [−3.849, −0.169], p = 0.032), but this association was attenuated after sport-domain adjustment in Model 3, consistent with extension ROM being more strongly influenced by broader anthropometric and sport-domain context than by SCM-specific morphology.

To address whether the pooled SCM CSA-function associations were consistent within sport domains, exploratory domain-stratified bivariate plots are presented in **Figure 2**. In the domain-stratified plots, maximal flexor strength showed a positive association with SCM CSA within every sport domain, although the association reached statistical significance only in Acceleration, Contact, and Para-athletes. Neck flexor endurance also showed positive within-domain slopes in all domains, with significant associations in Contact and Para-athletes. These plots are descriptive because several domain-specific samples were small, but they support the presence of within-domain SCM–function associations for the primary flexor outcomes.

#### 3.3.2 Neck girth models (model 1b: Neck girth + age + BMI)

Neck girth did not provide comparable explanatory value to SCM CSA for strength and endurance outcomes in adjusted models. For maximal flexor strength, neck girth was not associated with the outcome after age and BMI adjustment (β = +0.045, 95% CI [−0.274, +0.363], p = 0.781), whereas SCM CSA was significant in the corresponding Model 1a. For neck flexor endurance, neck girth was also non-significant (β = +0.094, 95% CI [−0.033, +0.221], p = 0.146). In contrast, neck girth showed a stronger relationship with extension ROM than SCM CSA (β = −2.094° per cm, 95% CI [−2.793, −1.395], p < 0.001), consistent with neck girth reflecting composite soft-tissue and body-size characteristics rather than muscle-specific functional capacity.

#### 3.3.3 Specificity models (model 2: SCM CSA + handgrip + age + BMI)

After adding handgrip strength (Model 2, n = 194), SCM CSA remained independently associated with maximal flexor strength (β = +1.245 kg per cm², 95% CI [+0.499, +1.991], p = 0.001; adjusted R² = 0.167) and neck flexor endurance (β = +0.517 min per cm², 95% CI [+0.212, +0.822], p < 0.001; adjusted R² = 0.072). In contrast, SCM CSA was not independently associated with maximal extensor strength in this model. These findings support a neck-specific association of SCM morphology with flexor strength and endurance that is not explained by general upper-limb strength capacity.

#### 3.3.4 Context models (model 3: SCM CSA + sport domain + age + BMI)

After adjustment for sport domain (Model 3), SCM CSA remained independently associated with maximal flexor strength (β = +1.347 kg per cm², 95% CI [+0.568, +2.126], p < 0.001; adjusted R² = 0.156) and neck flexor endurance (β = +0.582 min per cm², 95% CI [+0.277, +0.887], p < 0.001; adjusted R² = 0.127). A *post-hoc* exploratory within- versus between-domain decomposition further supported this interpretation: the within-domain SCM CSA component was significant for maximal flexor strength (β = +1.291, 95% CI [+0.508, +2.075], p = 0.001) and neck flexor endurance (β = +0.575, 95% CI [+0.262, +0.889], p < 0.001). The between-domain SCM CSA component was not significant for either outcome; however, this estimate was imprecise because it was based on only five domain means. Therefore, the main inference rests on the significant within-domain SCM component rather than on the null between-domain result. For maximal extensor strength, the higher adjusted R² in Model 3 reflected covariate and sport-domain contributions rather than an SCM-specific effect, as the semi-partial ΔR² for SCM CSA was only 0.007. For maximal extensor strength and extension ROM overall, SCM CSA effects were weaker or more strongly domain-related, supporting their interpretation as less SCM-specific outcomes.

## 4 Discussion

This study examined whether sternocleidomastoid (SCM) muscle morphology is associated with neck functional capacity in athletes and para-athletes, and whether these associations persist independently of age, BMI, general strength, and sport-domain context. The main findings were that SCM cross-sectional area showed a small-to-moderate association with maximal flexor strength and neck flexor endurance, and that these associations remained after adjustment for handgrip strength and sport domain. In contrast, SCM CSA was not independently associated with maximal extensor strength, and neck girth did not provide comparable explanatory value for strength or endurance outcomes. The adjusted R² values indicate low-to-modest explanatory power of the full models, and the semi-partial ΔR² values of approximately 5% for maximal flexor strength and neck flexor endurance indicate that SCM CSA adds measurable but limited unique explanatory information beyond age and BMI. Accordingly, ultrasound-based SCM assessment should complement, rather than replace, direct functional testing. For maximal extensor strength, the higher adjusted R² in the sport-domain model was driven by covariates and sport-domain context rather than by SCM CSA itself, consistent with the very small SCM-specific ΔR² for that outcome. This is relevant to the broader head–neck management literature, where cervical strengthening and greater neck strength have been discussed in relation to sport-related concussion risk, head-acceleration responses, neurostructural correlates, and sport-specific neck-strength profiles, particularly in collision and heading sports (Peek et al., 2020; Kavyani et al., 2021; de Souza et al., 2022; Leung et al., 2022; Alvacite Cardenas and Preston, 2025).

The differential findings between flexor and extensor strength are consistent with the anatomical role of the SCM as a neck flexor and anterior stabilizer, whereas maximal extensor strength is predominantly determined by posterior neck musculature not captured by SCM ultrasound. The observed SCM CSA-flexor strength association (r = 0.34) should therefore be interpreted as a small-to-moderate morphology-function relationship rather than as a strong structural determinant. Directionally, this finding is consistent with ultrasound-based cervical morphology research showing that neck muscle size and mechanical properties can be characterized by ultrasound and may differ across biologically relevant groups (Nagai et al., 2020). It is also compatible with previous sport-based strength research showing that neck strength differs across athletic and non-athletic populations (Rezasoltani et al., 2005). However, the magnitude of the present SCM CSA association is modest, and direct numerical comparison is limited because previous studies differed in population, neck muscle group, imaging site, and functional testing protocol.

Neck girth was not an independent predictor of flexor strength, extensor strength, or endurance after adjustment for age and BMI, despite small bivariate associations with flexor strength and endurance. This pattern suggests that neck circumference primarily reflects global body size rather than local cervical structural capacity, consistent with prior work questioning its utility as a functional proxy (Esopenko et al., 2020; Garrett et al., 2023).

The inverse association between neck size and extension ROM deserves specific consideration. Although SCM CSA showed only a weak inverse bivariate association with extension ROM, neck girth demonstrated a substantially stronger negative association with extension ROM. This suggests that reduced extension ROM is more closely related to global neck size and composite soft-tissue characteristics than to SCM morphology alone. Neck girth reflects muscle mass, adipose tissue, connective tissue, and overall body size, and may therefore mechanically limit neck extension or reflect sport-specific adaptations associated with larger neck dimensions. Importantly, this finding should not be interpreted as evidence that greater neck strength is harmful or that SCM hypertrophy directly reduces mobility. Rather, it highlights the need to assess neck strength, morphology, and ROM together, because greater structural capacity without adequate mobility may have implications for movement efficiency, functional performance, and potentially injury risk.

The domain-stratified plots and *post-hoc* within- versus between-domain analysis help reconcile the pooled and adjusted findings. The domain-stratified plots showed positive within-domain SCM CSA associations with maximal flexor strength in all sport domains, although some domain-specific estimates were non-significant because of small sample sizes. In the decomposition analysis, the within-domain SCM CSA component remained significant for maximal flexor strength and neck flexor endurance. The between-domain component was not significant for these two outcomes, but this null result should be interpreted cautiously because it was estimated across only five domains and was therefore imprecise. Thus, the main support for a morphology-function relationship comes from the significant within-domain SCM component rather than from absence of a between-domain effect. Conversely, maximal extensor strength and extension ROM appeared more strongly influenced by broader sport-domain or anthropometric context, consistent with their weaker SCM-specific interpretation.

Para-athletes showed a distinct cervical functional profile, characterized by lower maximal flexor and extensor strength and reduced extension ROM compared with non-para participants, while neck flexor endurance was similar. These findings should be interpreted with caution because the para-athlete subgroup was small (n = 20) and heterogeneous, including several para-sport disciplines and impairment profiles. Nevertheless, the pattern highlights the importance of considering impairment-related constraints, testing context, and sport-specific demands when interpreting cervical morphology and functional capacity in para-athletes.

Future studies should examine the interaction between neck morphology, strength, and mobility using longitudinal designs. In particular, training studies should evaluate whether increases in SCM CSA and neck strength occur with preserved or reduced neck ROM, and whether combined strengthening and mobility interventions provide more favorable functional profiles than strengthening alone. Future research should also include female athletes, younger athletes, and larger sport-specific cohorts, extend ultrasound-based assessment to posterior neck muscles, and link morphology–function profiles with injury surveillance, pain, head-impact exposure, and sport-specific performance outcomes.

### 4.1 Limitations

The sample included adult male athletes and para-athletes across multiple sport domains, which may limit generalizability to younger athletes, female athletes, and clinical populations. The para-athlete subgroup was small and heterogeneous, spanning several para-sport disciplines and impairment profiles, and therefore para-athlete-specific findings should be interpreted cautiously. The cross-sectional design prevents causal inference, and the absence of inter-rater reliability data for the ultrasound protocol is a further limitation. Although the *post-hoc* within- versus between-domain analysis helped address the possibility of aggregation effects, several within-domain estimates were based on modest subgroup sample sizes, and the between-domain estimates were imprecise because only five domains were available. Future studies should incorporate longitudinal designs, larger sport- and impairment-specific cohorts, and ultrasound assessment of posterior neck muscles to evaluate whether changes in SCM morphology predict changes in neck function and clinically relevant outcomes.

## 5 Conclusions

SCM cross-sectional area was independently associated with maximal neck flexor strength and neck flexor endurance in athletes and para-athletes after adjustment for age, BMI, handgrip strength, and sport domain. These associations reflected modest within-domain structure-function coupling rather than only between-domain aggregation, although between-domain estimates were imprecise because of the small number of domains. Neck girth was not an independent predictor of strength or endurance outcomes after adjustment, suggesting that circumference-based anthropometry is an insufficient proxy for local neck structural capacity. These findings support musculoskeletal ultrasound assessment of SCM morphology as a clinically informative adjunct to objective neck functional testing, while also emphasizing that SCM morphology explains only part of cervical performance and should be interpreted alongside sport context, general strength, and mobility.

## Data Availability

The raw data supporting the conclusions of this article will be made available by the authors, without undue reservation.
